# How coronavirus survives for days on surfaces

**DOI:** 10.1063/5.0033306

**Published:** 2020-11-01

**Authors:** Rajneesh Bhardwaj, Amit Agrawal

**Affiliations:** Department of Mechanical Engineering, Indian Institute of Technology Bombay, Mumbai 400076, India

## Abstract

Our previous study [R. Bhardwaj and A. Agrawal, “Likelihood of survival of coronavirus in a respiratory droplet deposited on a solid surface,” Phys. Fluids **32**, 061704 (2020)] showed that the drying time of typical respiratory droplets is on the order of seconds, while the survival time of the coronavirus on different surfaces was reported to be on the order of hours in recent experiments. We attribute the long survival time of the coronavirus on a surface to the slow evaporation of a thin nanometer liquid film remaining after the evaporation of the bulk droplet. Accordingly, we employ a computational model for a thin film in which the evaporating mass rate is a function of disjoining and Laplace pressures inside the film. The model shows a strong dependence on the initial thickness of the film and suggests that the drying time of this nanometric film is on the order of hours, consistent with the survival time of the coronavirus on a surface, seen in published experiments. We briefly examine the change in the drying time as a function of the contact angle and type of surface. The computed time-varying film thickness or volume qualitatively agrees with the measured decay of the coronavirus titer on different surfaces. The present work provides insights on why coronavirus survival is on the order of hours or days on a solid surface under ambient conditions.

The role of respiratory droplets in spreading COVID-19 has already been well-documented.^1–7^ These droplets, produced during coughing, sneezing, and moist speaking, form fomites upon falling on a surface, which could be a source of secondary infection. Fecal-oral route for the transmission of SARS-CoV-2 (referred to as coronavirus, hereafter) has also been examined recently.^8^ Understandably, the survival of the coronavirus in such infected droplets and its duration are of paramount interest,^9,10^ which has led to research on the nature of drying of droplets on various surfaces and factors affecting the drying time. For instance, Bhardwaj and Agrawal^3^ examined the drying time of droplets using a diffusion-limited model. They found that for droplets of volume of 1 nl to 10 nl (corresponding to the diameter of 125 *μ*m–270 *μ*m), produced during coughing/sneezing/speaking,^11^ the drying time on a surface (with a contact angle of 10°–90°) varies between 2 s and 137 s, respectively. Any convection in the room will reduce the drying time further. In a follow-up study,^4^ they showed that the drying time is a non-monotonic function of the contact angle. The drying time increases with contact angle up to 148° and decreases slightly for 148°–179°.

To understand the survival of the coronavirus on a solid surface, Chin *et al.*^10^ examined the survival time of the SARS-CoV-2 virus on different surfaces and at different temperatures, pH, and other conditions. The mean log reduction in titer for glass was reported to be 3.39 fifty-percent tissue culture infective dose (TCID_50_)/ml over a 2-day duration, while for plastic, the corresponding reduction was 3.54 TCID_50_/ml over 4 days. Similarly, a long survival time of the coronavirus on different surfaces was reported by von Doremalen *et al.*^9^ An interaction between several respiratory droplets deposited on a surface can increase the drying time; however, it is unlikely that the drying time would increase to hours and days, required for the survival of the virus. While the initial virus concentration considered in the above studies^9,10^ is more than what is realistically expected,^12^ the question of the long survival time of the virus on surfaces remains unanswered. Here, we show that this discrepancy can be resolved by considering the effect of disjoining pressure, which becomes particularly important when the film thickness reduces to a few nanometers. Although this nanometric film has a negligible volume compared to the initial volume of the droplet, the film is large enough compared to the size of the virus and provides enough medium required for the long survival of the virus. That is, while the evaporation of a large volume of the droplet (>99.9%) occurs in a few minutes as suggested by the earlier calculations,^3,4^ the evaporation of the remaining volume takes far longer (as shown in this work), and the virus can survive in this remaining volume.

An excess free energy exists in the liquid film due to long-range, dispersive forces between solid–liquid and liquid–vapor (LV) interfaces. This leads to a net repulsive force per unit area between the interfaces, known as disjoining pressure.^13–17^ The disjoining pressure varies inversely as the cube of the film thickness, and the proportionality constant is known as Hamaker constant. Given the low value of the Hamaker constant (of the order of 10^−20^ J), the disjoining pressure becomes relevant only for sub-micron thickness films. The effect of disjoining pressure has been considered in earlier studies on wetting^18,19^ and evaporation.^20–22^ For example, Ajaev^23^ incorporated the effect of disjoining pressure for both perfectly wetting and partially wetting liquids. The evolution of film thickness as a function of space and time was solved, from where other quantities of interest can be derived. Therefore, the primary objective of the present work is to explain the long survival time of the coronavirus on surfaces. We hypothesize that the liquid film with thickness on the order of sub-micrometers or nanometers that remains on the surface, due to the London–van der Waals forces, continues to provide a hotbed for the survival of the virus, even after the disappearance of the bulk of the droplet owing to evaporation. We compute the drying time of droplets due to Laplace and disjoining pressures and find the time to be on the order of hours, thereby rendering credence to our hypothesis.

First, we present a computational model to estimate the drying time of a thin liquid film evaporating on a solid surface. Initially, a respiratory droplet deposits as a spherical cap on the surface (Fig. 1), whose volume (*V*) and equilibrium contact angle (*θ*_*E*_) are expressed as follows:

$$V=\frac{\pi H}{6}\left(3R^{2}+H^{2}\right),\theta_{E}=2\tan^{-1}\frac{H}{R},$$ (1)

where *H* and *R* are the droplet height and wetted radius, respectively. As the droplet evaporates, the droplet becomes a thin film with a thickness on the order of sub-micrometers. We consider a pancake-like thin film that partially wets the surface with contact angle *θ*_*E*_ and thickness *h*_0_ (Fig. 1) in cylindrical coordinates. The film is considered with a pinned contact line throughout the evaporation. The pinning could be due to roughness or impurities (e.g., dust) on the surface. The approximate film volume at a given time-instance is expressed as follows:

$$V=\pi R^{2}h,$$ (2)

where *h* is the film thickness and the initial volume of the film is *V*_0_ = *πR*^2^*h*_0_. The disjoining pressure in the film (Π, N/m^2^) as a function of the film thickness (*h*) is given by^16,17^

$$\Pi \left(h\right)=\frac{A_{H}}{6\pi h^{3}},$$ (3)

where *A*_*H*_ is the Hamaker constant (Joules). The Hertz–Knudsen law describes the evaporative mass flux *J* [kg/m^2^ s] of a thin liquid film into its saturated vapor using kinetic theory of gases. The expression of *J* is given by^20,21,23–25^

$$J=\frac{\rho_{V}}{\sqrt{2\pi RT_{sat}}}\left[\frac{1}{\rho_{L}}\left(P-P_{V}\right)+\frac{La}{T_{sat}}\left(T_{LV}-T_{sat}\right)\right],$$ (4)

where *P* is the pressure in the liquid film, R is the ideal gas constant per unit mass, *T*_*sat*_ is the liquid saturation temperature, *La* is the latent heat of vaporization per unit mass, *T*_*LV*_ is the temperature of the liquid–vapor (LV) interface, and *ρ*_*V*_ and *ρ*_*L*_ are the densities of the liquid and liquid vapor, respectively. We consider the evaporating film and substrate at ambient temperature (*T*_*amb*_) and neglect the temperature drop across the film thickness. This is justified for a thermally conductive substrate and if the ratio of the thickness of the film to the substrate is very small. The liquid vapor is in the saturated state (i.e., 100% relative humidity) just above the liquid–vapor interface. Therefore, *T*_*LV*_ ≈ *T*_*sat*_ ≈ *T*_*amb*_ and the last term in Eq. (4) is zero. Equation (4) simplifies to

$$J=\frac{\rho_{V}}{\rho_{L}\sqrt{2\pi RT_{amb}}}\left(P-P_{V}\right).$$ (5)

The pressure inside the film is given by using the augmented Young–Laplace equation^20,22,23^

$$P-P_{V}=\Pi \left(h\right)-\frac{\gamma \frac{d^{2}z}{dr^{2}}}{{\left[1+{\left(\frac{dz}{dr}\right)}^{2}\right]}^{3/2}},$$ (6)

where the second term denotes the Laplace pressure in the film and *γ* is the surface tension of the film with respect to air. Using order of magnitude analysis, the derivatives in Eq. (6) are approximated as *d*^2^*z*/*dr*^2^ ∼ *h*/*R*^2^ and *dz*/*dr* ∼ *h*/*R*. Since *h*/*R* ≪ 1, we approximate *dz*/*dr* ≈ 0. Therefore, using this approximation and Eq. (3), the pressure inside the film is given by

$$P-P_{V}=\frac{A_{H}}{6\pi h^{3}}-\frac{\gamma h}{R^{2}}.$$ (7)

**FIG. 1.**
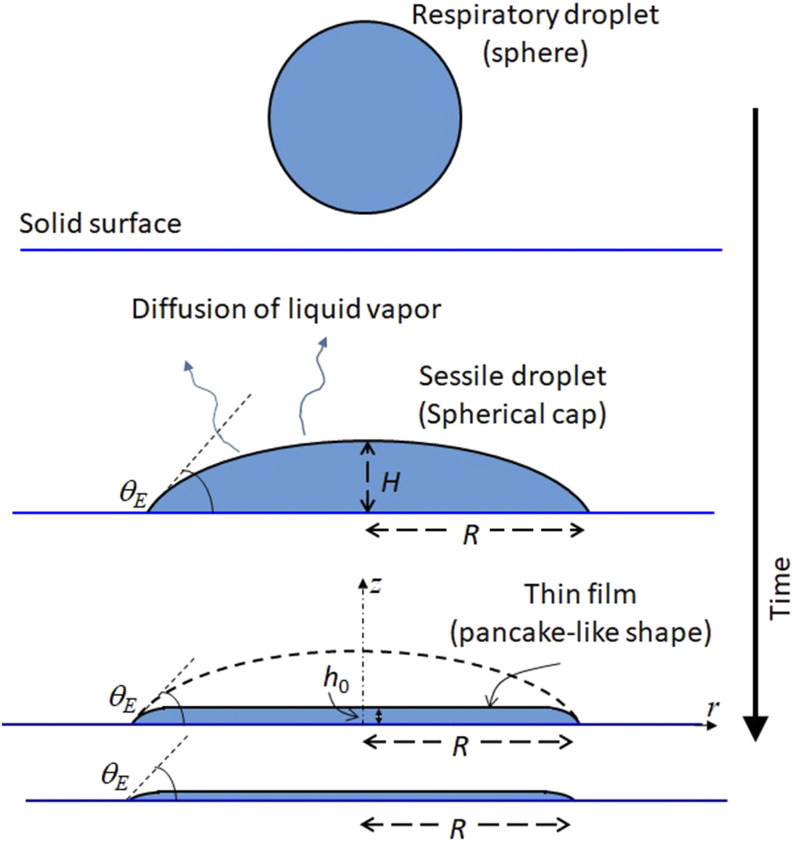
Schematic of the problem considered in the present study. A respiratory droplet deposits on a surface as a sessile spherical cap and evaporates by diffusion of liquid vapor in air. The present study focuses on the evaporation of the thin film that forms in the later stages of the evaporation.

Using Eq. (5), the expression of evaporative mass flux is as follows:

$$J=\frac{\rho_{V}}{\rho_{L}\sqrt{2\pi RT_{amb}}}\left[\frac{A_{H}}{6\pi h^{3}}-\frac{\gamma h}{R^{2}}\right].$$ (8)

Since *A*_*H*_ < 0 for a water film on solid surfaces considered here (Table I), the disjoining and Laplace pressures both contribute to *J*. Equation (8) shows that *J* scales as *A*_*H*_, and since *R* is constant throughout the evaporation, *J* inversely scales with *R*.

**TABLE I.** Parameters for the cases considered for comparison of reduction in titer of coronavirus on a surface with model predictions in the present study. Hamaker constants are listed for the substrate (medium 1) interacting with air (or vacuum, medium 2) across water (medium 3). The thickness of the adsorbed film (*h*_*ad*_) is calculated using Eq. (16). The sources of the data utilized from the literature are given in the last two columns. *A*_132_ is computed using the combining relation^26,27^ $A_{132}\;\approx \;\left(\sqrt{A_{11}}-\sqrt{A_{33}}\right)\left(\sqrt{A_{22}}-\sqrt{A_{33}}\right)$, where *A*_33_ = 3.7 × 10^−20^ J and *A*_22_ = 0.

System (1–3–2)	*A*_11_ (J)	*A*_132_ or *A*_*H*_ (J)	*θ*_*E*_ (deg)	*h*_*ad*_ (Å)	Source of A_11_	Source of *θ*_*E*_
Glass–water–air	6.8 × 10^−20^	−1.3 × 10^−20^	29	3.4	Ref. 26	Ref. 28
Copper–water–air	40.2 × 10^−20^	−8.5 × 10^−20^	70	3.8	Refs. 26 and 27	Ref. 29
Polypropylene–water–air	5.1 × 10^−20^	−0.7 × 10^−20^	84	0.9	Ref. 26	Ref. 30
Stainless steel–water–air	21.2 × 10^−20^	−5.2 × 10^−20^	32	6.2	Ref. 31	Ref. 32

Furthermore, we derive an ordinary differential equation for computing the drying time of the film. The mass loss rate (kg/s) from the film in terms of *J* is expressed as follows:

$$\rho_{L}\frac{dV}{dt}=\int_{0}^{R}2\pi r\sqrt{1+{\left(\frac{dz}{dr}\right)}^{2}}Jdr.$$ (9)

Substituting Eq. (2) and *dz*/*dr* ≈ 0 (as shown earlier) in Eq. (9), we rewrite Eq. (9) as follows:

$$\frac{dh}{dt}=\frac{J}{\rho_{L}}.$$ (10)

From Eqs. (8) and (10), the time-derivative of film thickness is given by

$$\frac{dh}{dt}=\frac{\rho_{V}}{\rho_{L}^{2}\sqrt{2\pi RT_{amb}}}\left[\frac{A_{H}}{6\pi h^{3}}-\frac{\gamma h}{R^{2}}\right].$$ (11)

Eq. (11) is solved using the fourth-order Runge–Kutta method. A time step of 0.5 s is used after conducting a time step independence study. The film continues to evaporate until its thickness reaches a critical value (*h*_*ad*_), and it does not evaporate further since it is adsorbed on the surface in this equilibrium state.

We estimate the thickness of the adsorbed film (*h*_*ad*_) as follows. In the equilibrium state, the excess energy per unit area of the film (*E*, J/m^2^) and disjoining pressure (Π, N/m^2^) in the film are expressed as a function of the spreading parameter, *S*, as follows:^17^

$$S=E\left(h_{ad}\right)+h_{ad}\Pi \left(h_{ad}\right),$$ (12)

and *S* for the partial wetting regime is defined as follows:^17^

$$S=-\gamma \left(1-\cos\theta_{E}\right).$$ (13)

*E* is a function of Π, given by^17^

$$\Pi \left(h\right)=-\frac{dE}{dh}.$$ (14)

Using Eq. (3), integrating Eq. (14), and applying *E*(*∞*) = 0, *E* is expressed in terms of *h* as follows:^17,19^

$$E=\frac{A_{H}}{12\pi h^{2}}.$$ (15)

Using Eqs. (3), (12), (13), and (15), we obtain the following equation:

$$-\gamma \left(1-\cos\theta_{E}\right)=\frac{A_{H}}{4\pi h_{ad}^{2}}.$$ (16)

We utilize Eq. (16) to obtain the thickness of the adsorbed film (*h*_*ad*_), and the simulations are stopped when the film thickness computed from Eq. (11) reduces to *h*_*ad*_. We present simulations for water films on different surfaces, formed by 5 *μ*l and 50 *μ*l droplets. The parameters used in the model are listed in Tables I–III.

**TABLE II.** Thermophysical properties and values of constants used in the present simulations.

Property/condition	Value
Ambient temperature, *T*_*amb*_	298 K
Surface tension of water film, *γ*	0.072 N/m
Specific gas constant for water vapor, R	461.5 J/kg K
Density of water, *ρ*_*L*_	1000 kg/m^3^
Density of water vapor saturated at T_*amb*_, *ρ*_*V*_	0.023 kg/m^3^

**TABLE III.** Wetted radius of droplets estimated using Eq. (1) for droplets of 5 *μ*l and 50 *μ*l on different surfaces.

Substrate	*V* (*μ*l)	*R* (mm)
Glass	5	2.3
Copper	50	3.4
Polypropylene	5	1.4
Polypropylene	50	3.0
Stainless steel	5	2.2
Stainless steel	50	4.8

Second, we present the time variation of the film thickness during the evaporation, employing the model presented. We consider a 5 *μ*l water droplet on glass, with a contact angle of 29°. The parameters for carrying out the computation are listed in Table I. The time variation of the thickness of the film for an initial film thickness of *h*_0_ = 400 nm is plotted in Fig. 2(a). Since the volume of the film scales linearly with its thickness [Eq. (2)], the time variation of the volume is the same as that of the thickness. The time taken for evaporation for this case is 84 h, and the film thickness reaches *h*_*ad*_ at the end of evaporation. The time variation of the thickness is linear for around 60% decrease in the initial thickness of the film, which takes around 83% of the total drying time. The thickness variation is non-linear for the rest of the evaporation. The time variation of the disjoining and Laplace pressures is compared in Fig. 2(b). At the commencement of the evaporation, the disjoining pressure is around two times the Laplace pressure, while the former becomes roughly one order of magnitude larger than the latter at around 70 h. This trend can be explained using Eq. (7) since these pressures evolve as *h*^−3^ and *h*, respectively, where *h* is the film thickness. Therefore, the evaporation mass flux is dominated by disjoining pressure during the evaporation and the non-linear decay of the thickness is attributed to an exponential increase in the disjoining pressure in the last stage of the evaporation.

**FIG. 2.**
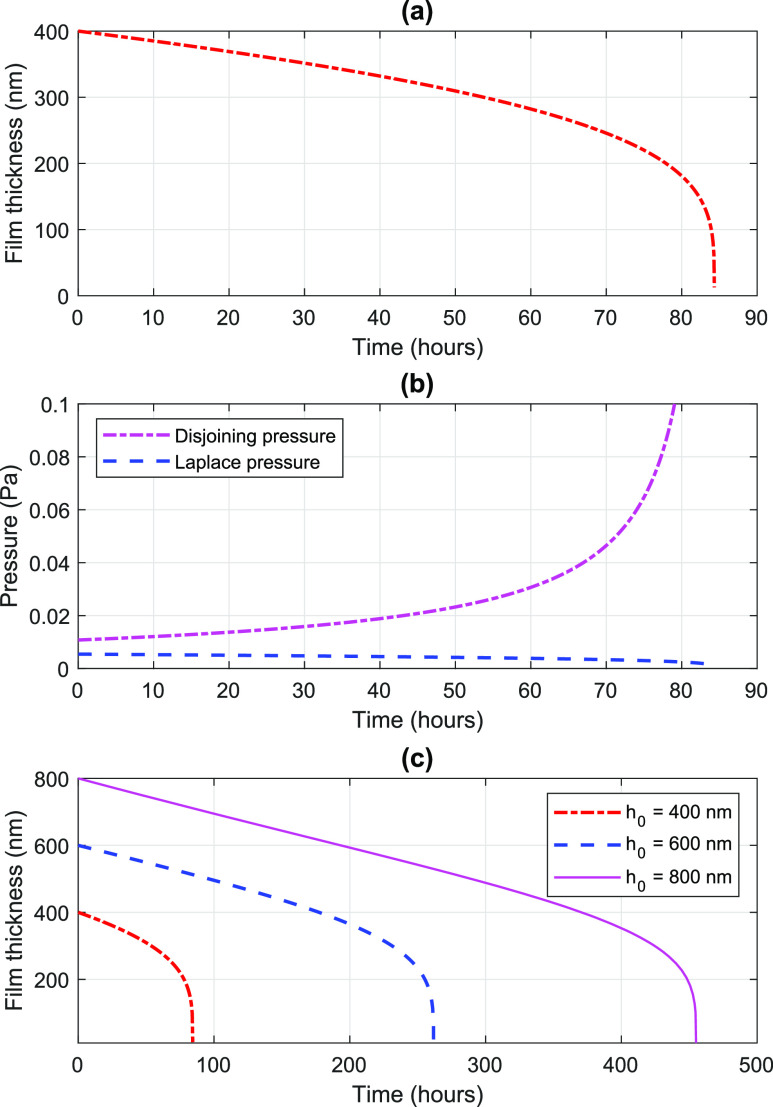
(a) Time-varying film thickness of a water film on a glass surface of initial film thickness *h*_0_ = 400 nm. (b) Evolution of disjoining and Laplace pressures inside the film. (c) Comparison among the time-varying film thickness of a water film on a glass surface for three cases of initial film thickness: *h*_0_ = 400 nm, 600 nm, and 800 nm.

Figure 2(c) compares the time variation of the film thickness for three cases of the initial film thickness: *h*_0_ = 400 nm, 600 nm, and 800 nm. The respective drying times are 84 h, 262 h, and 455 h, exhibiting a linear increase in the drying time with the initial film thickness or initial volume. While the drying time is larger for a larger thickness, the time variation is linear for around 85%–90% of the drying time for all the cases. The slope of linear decay is almost the same in all cases.

To compare and contrast, we compute the drying time of a 5 *μ*l water droplet on glass using the diffusion-limited model presented in our previous work.^3^ The drying time is around 28 min (considering 50% relative humidity). Thus, the two disparate time scales obtained in the two models show that the diffusion-limited model does not apply to the last stage of drying, i.e., when the droplet evolves to a thin film on the surface. Thus, this model grossly underestimates the final drying time of the droplet since the residual film evaporates much slower than the remaining droplet.

Third, we describe cases of water film evaporation on different surfaces, namely, glass, copper, polypropylene, and stainless steel. 5 *μ*l and 50 *μ*l water droplets on these surfaces are considered. These combinations of droplet volumes and surfaces were used in very recent experiments on measuring the titer of the coronavirus.^9,10^ The parameters used for these computations are given in Tables I–III. We compute the temporal evolution of the film thickness using the model presented earlier and compare it with the reduction in the virus titer measurements^9,10^ in Fig. 3. The frames in the left and right columns show the comparison with virus titer obtained using 5 *μ*l (Ref. 10) and 50 *μ*l (Ref. 9) droplets, respectively. The drying time of the film on glass, polypropylene, and stainless steel for a 5 *μ*l droplet is 84 h, 96 h, and 24 h respectively, as shown in the frames in the left column in Fig. 3. The ratio among these times inversely scales with the ratio among the respective Hamaker constants of these surfaces (Table I). A similar trend is noted for the drying times for a 50 *μ*l droplet. The drying time does not exhibit a strong dependence on the wettability (or contact angle) of the surface. For example, the film drying is the fastest on copper and the slowest on polypropylene for a 50 *μ*l droplet (Fig. 3). However, the contact angle of these two cases is almost similar (Table I). Since the Hamaker constant of copper is one order of magnitude larger than that of polypropylene (Table I), the drying time is shorter on the former as compared to the latter. Therefore, the Hamaker constant is a more important parameter than the wettability of the surface to determine the drying time of the film.

**FIG. 3.**
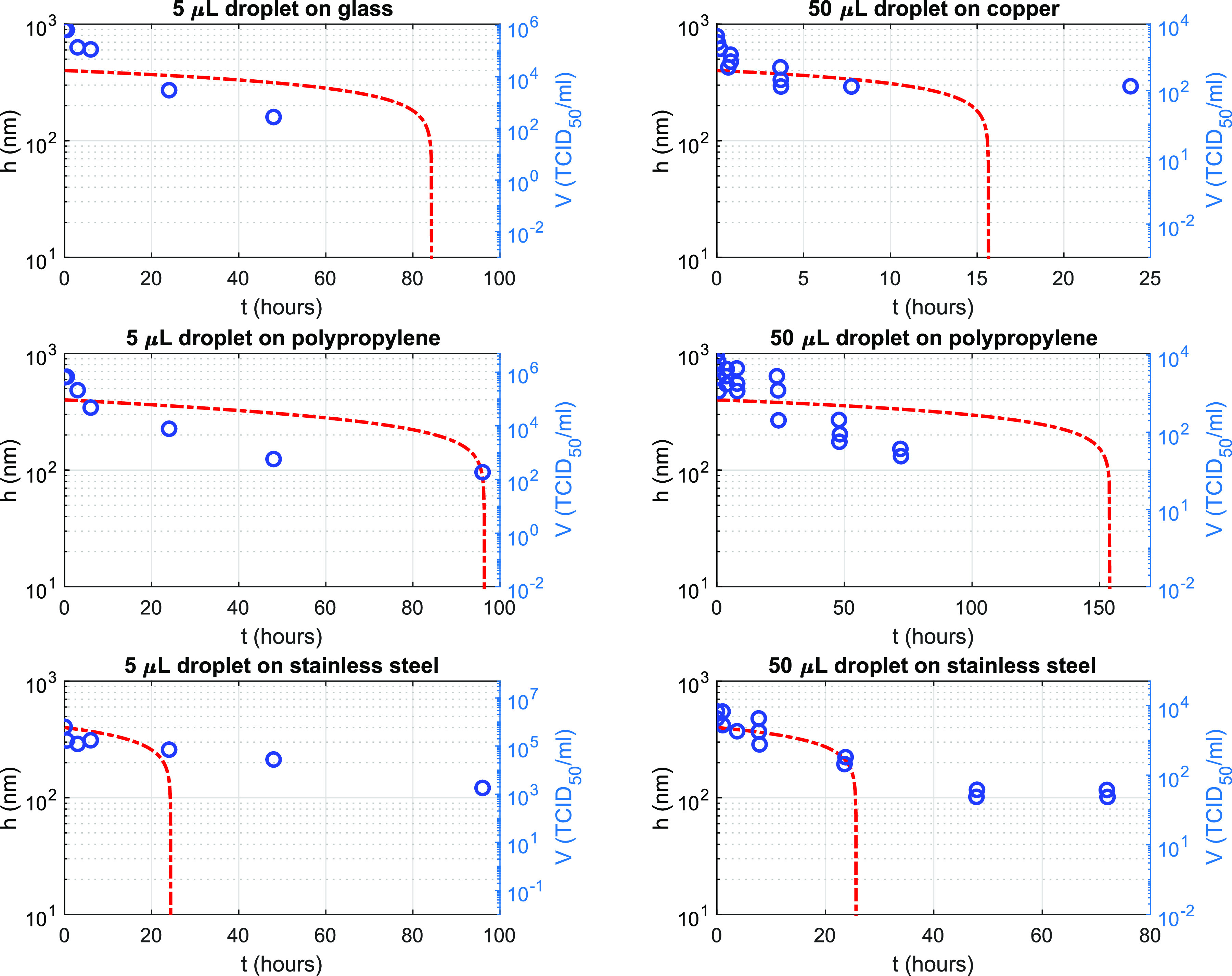
Time-varying evaporating film thickness (*h*, red dashed line) and virus titer (*V*, blue circles) plotted on the left and right y axes, respectively. The frames in the left and right column correspond to 5 *μ*l and 50 *μ*l droplets, respectively. Virus titer for four different surfaces, namely, glass, copper, plastic (polypropylene), and stainless steel, are plotted, as reported in recent experiments.^9,10^ The initial film thickness in simulations is taken as 400 nm in all cases.

We further compare the reduction in virus titer with time-varying film thickness (or volume) in Fig. 3. The slope of the reduction in virus titer with time matches qualitatively well with the thickness change with time of the film for all cases. The drying time in the cases of glass, copper, polypropylene, and stainless steel is on the same order as that of the respective time when the titer stops changing with time. The model slightly underpredicts this time for the case of stainless steel for a 5 *μ*l droplet. Overall, the evolution of the film thickness matches with time-varying virus titer recorded in the measurements.^9,10^

Finally, we discuss the relevance of the present results to the spread of COVID-19 by respiratory droplets on a surface (i.e., fomite). While the evaporation of a microliter droplet predicted by a diffusion-limited model is on the order of minutes, the virus titer survival is on the order of hours and days reported in recent measurements. The present model for the film evaporation shows that the survival or drying time of a thin liquid film on a surface is also on the order of hours and days, similar to what has been observed in measurements of the virus titer. Therefore, it is likely that the virus survives in the liquid film, which evaporates much slower than the major part of the volume of the droplet. These observations are also consistent with an earlier study,^33^ in which the reduction in virus titer was correlated with the drying of an aqueous droplet, laden with 19 different viruses, on a glass surface. An increase in temperature, which helps dislodge this nanometric film, reduces the survivability of the virus on the surface. This has been confirmed by the experiments of Chin *et al.*^10^ where the virus was undetected after 1 min of keeping the substrate at 70 °C.

The coronavirus titer in recent experiments has been shown to change with the surface. In general, the virus survival time is shorter on metals as compared to plastic or glass. For instance, the survival of the coronavirus on copper is shorter than that on polypropylene. The model for the drying time of the film shows the same trend and qualitatively validates the hypothesis that a longer virus survival is due to the thin film present on the surface. The thickness of the adsorbed film on the surface (Table I) is on the order of Å, three orders of magnitude lesser than the diameter of the coronavirus (≈120 nm). Hence, it is unlikely that the coronavirus can survive in the adsorbed film on the surface. Therefore, the drying of the film will obliterate the virus eventually. Since a longer survival time of the virus corresponds to larger chances of the infection of COVID-19, it is desirable to disinfect frequently touched surfaces (e.g., a door handle) or surfaces in areas prone to outbreaks (e.g., common areas in a hospital). We also recommend heating the substrate wherever possible, as even short-duration high-temperature heating helps evaporate the nanometric film and disintegrates the virus. These measures will help remove the residual liquid film on these surfaces, thereby reducing the chances of infection. The frequency of cleaning of surfaces should account for the longer drying times of the film, as predicted by the model in the present work. Finally, it is safer to employ metal surfaces rather than plastic since the survival of the coronavirus is shorter on the former.

There are a few limitations of the model presented here, which can be addressed in future studies. We have assumed a water film while the residual film of saliva or mucus respiratory droplets may contain solute. The drying time of a film with biological solute would be longer, as shown earlier for droplet drying^34^ and has been explained by Raoult’s effect. The drying time of a saliva droplet is around 25% longer as compared to a pure water droplet deposited on a surface.^34^ Therefore, the uncertainty in the present results due to neglecting solute in the model is not significant. Furthermore, the present model cannot tackle the evaporation of a liquid film on porous surfaces such as cardboard and textiles. The droplet would impregnate a porous surface, and the drying of the liquid film on such a surface is expected to be faster. However, this situation is not conducive to the survival of the virus, and the virus titer on cardboard and textiles has been shown to decay faster as compared to that on metal and plastic surfaces.^9,10^ Therefore, it is further reaffirmed that the virus survives in the liquid film present on the surface. Finally, a liquid film on an inclined surface could exhibit gravity-driven flow inside the film, which has not been accounted for in the present work.

In closure, we have developed a computational model based on kinetic theory to compute the time-varying thickness and drying time of a liquid film on a solid surface. The computed drying time of a film with an initial thickness on the order of nanometers is on the order of hours and days. The drying time is, however, on the order of seconds or minutes for a nanoliter or microliter droplet, respectively, predicted by a diffusion-limited evaporation model. The evolution of the film thickness is compared with the time-varying reduction in the titer of the coronavirus reported in two recent studies on different surfaces. The survival times of the coronavirus are consistent with the times of the evaporation of a liquid film found in the present work. Moreover, the trends of time variation of film thickness (or volume) qualitatively agree with the reduction in the titer of the coronavirus in published measurements. The model captures the relatively shorter survival times on metals as compared to that on plastic and glass, explained by the larger value of the Hamaker constant of the metal–water–air system. Our hypothesis is also consistent with the effect of temperature seen in previous experiments. Overall, the present computations are the first attempt to show that the film evaporation time is on the order of hours/days, and these results explain the experimentally observed longer survival time of the coronavirus on a surface. These results also highlight the need for cleaning of frequently touched surfaces and suggest that even short-duration high-temperature heating of surfaces can reduce the chances of the virus survival and infection of COVID-19.

## DATA AVAILABILITY

The data that support the findings of this study are available from the corresponding author upon reasonable request.
